# From Determinism and Probability to Chaos: Chaotic Evolution towards Philosophy and Methodology of Chaotic Optimization

**DOI:** 10.1155/2015/704587

**Published:** 2015-03-24

**Authors:** Yan Pei

**Affiliations:** Computer Science Division, The University of Aizu, Tsuruga, Ikki-machi, Aizu-Wakamatsu, Fukushima 965-8580, Japan

## Abstract

We present and discuss philosophy and methodology of chaotic evolution that is theoretically supported by chaos theory. We introduce four chaotic systems, that is, logistic map, tent map, Gaussian map, and Hénon map, in a well-designed chaotic evolution algorithm framework to implement several chaotic evolution (CE) algorithms. By comparing our previous proposed CE algorithm with logistic map and two canonical differential evolution (DE) algorithms, we analyse and discuss optimization performance of CE algorithm. An investigation on the relationship between optimization capability of CE algorithm and distribution characteristic of chaotic system is conducted and analysed. From evaluation result, we find that distribution of chaotic system is an essential factor to influence optimization performance of CE algorithm. We propose a new interactive EC (IEC) algorithm, interactive chaotic evolution (ICE) that replaces fitness function with a real human in CE algorithm framework. There is a paired comparison-based mechanism behind CE search scheme in nature. A simulation experimental evaluation is conducted with a pseudo-IEC user to evaluate our proposed ICE algorithm. The evaluation result indicates that ICE algorithm can obtain a significant better performance than or the same performance as interactive DE. Some open topics on CE, ICE, fusion of these optimization techniques, algorithmic notation, and others are presented and discussed.

## 1. Introduction

Philosophy of determinism comes from the development of classic mechanic that was originally established and studied by Isaac Newton, Pierre-Simon Laplace, Gottfried Wilhelm Leibniz, and so forth. Strict determinism indicates that causality can be expressed and implemented by mathematical calculation and logical reasoning. As Pierre-Simon Laplace said, “we may regard the present state of the universe as the effect of its past and the cause of its future” [1]. In the philosophy of determinism, everything is deterministic and predictable. However, the discovery of probability breaks dominated position of determinism in scientific philosophy. Particularly, the proposal of law of larger numbers extends the recognition scale of science, which explains the relationship between probability and frequency. The two philosophies and methodologies, determinism and probability, dominate researches in science until the discovery of chaos, another philosophy and methodology which can present and explain the natural world. In optimization field, deterministic and stochastic optimization algorithms are theoretically supported by philosophy and methodology of determinism and probability. However, because some fundamental works of chaos theory are not completed yet, research and development of chaotic optimization algorithm are still rarely mentioned and studied in optimization field. This paper tries to present a limit work on chaotic optimization algorithm with evolution concept.

Most of evolutionary computation (EC) algorithms are inspired from natural phenomena, such as genetic algorithm that mimics the process of natural selection and survival of the fittest. EC can be involved in continuous and combinational optimization problems, and its algorithm has a metaheuristic or stochastic characteristic in their search mechanisms [2]. As EC algorithms have been developed further and deeply, not only biological phenomena but also physical and mathematical phenomena and mechanisms are introduced into EC area to implement new EC algorithms. This research subject is one of computational paradigm studies in natural computing area and enriches research context of computational intelligence. In a viewpoint of EC search scheme, its algorithms encompass two components in their search mechanisms. One is a search methodology by a variety of implementations, and the other is an iterative process to simulate evolution. Most of EC algorithms do not require the optimization problem to have some specific characteristics, and few of them utilize any mathematical properties or mechanisms to ensure convergence of the algorithms. If we introduce mathematical optimization property or mechanism that simulates the natural phenomena, such as chaotic ergodicity, into an optimization iterative process, we may implement new EC algorithms that partially ensure their convergence. The hint and philosophy behind this motivation benefit are to improve the global convergence characteristic of the algorithm.

The study on chaos theory comes from the real problems of physics, ecology, and mathematics since three-body problem was studied by Poincare et al. [3, 4]. It is developed originally to describe the system behaviour that cannot be described either by deterministic system or by stochastic system, which enriches our knowledge on unpredictable property of natural system. That means the system cannot be normalized by a set of differential equations or probability density function. Chaos and chaotic system have many characteristics for implementing an evolutionary search prototype and framework and improving EC algorithms. Nonlinearity, ergodicity, and sensitiveness are major explicit properties of chaos and chaotic system. The ergodicity of chaotic system can support more diversity to enhance exploration and exploitation capability of EC algorithms. It can approach to any desired point in search space with arbitrary accuracy and movement track. The sensitiveness of initial condition of chaotic system can lead to different search paths and escape from local optima for enhancing search performance of EC algorithms. The motion of perturbation around chaotic attractor can be simulated as an optimization search scheme and framework by EC.

There are typically three applications in which chaos is used in optimization area, that is, a local search method, a parameter tuning technique, and the new EC algorithm inspiration resource. Some chaotic systems are introduced into conventional EC algorithm frameworks to make population diversity in local search and to tune the parameters of algorithm [5]. The ergodicity property and behaviour are modelled as a search prototype to make a new EC algorithm, such as chaotic evolution optimization framework [6]. Chaotic Krill Herd algorithm was proposed by fusing Krill Herd algorithm and chaos theory [7], and some of its improved versions were proposed and studied [8, 9].

Chaotic evolution (CE) is a population-based algorithm framework that simulates chaotic motion behaviour in a search space [6]. Because of the ergodicity of chaotic system, chaotic motion can visit any point with arbitrary accuracy. CE algorithms take into account ergodicity property in its search mechanism to implement a new optimization scheme. There are three parameters as algorithm setting in its framework, a chaotic system and its parameter(s), a direction rate to guide the percentage of search direction, and a crossover rate. The chaotic system supports a basic simulation parameter, chaotic parameter, to implement the search function. Parameter of the chaotic system is simply set at the value that can lead to a chaotic output of the system. The direction rate decides search direction of each individual in CE algorithm, which is usually set at a random value. The crossover operation reserves extensibility of CE algorithm for further study, which can be set at 100%. It means that the mutant vector of CE algorithm can be directly presented as the chaotic vector of CE algorithm. For an empirical study, it is not a necessary parameter in CE optimization framework [6]. However, we cannot deny its effectiveness in theory from our current best knowledge.

This paper extends the work of [6] by introducing new chaotic systems into the CE algorithm framework and conducts a comparison study with DE algorithms. We use 25 benchmark functions with 10- and 30-dimensional setting to evaluate our new designed CE algorithms. We also establish a new interactive EC (IEC) algorithm by using CE optimization framework and evaluate its performance by using a pseudo-IEC user, that is, a Gaussian mixture model. Some statistical tests, such as Wilcoxon sign-ranked test, Friedman test, and Bonferroni-Dunn's test, are applied to evaluate significance and ranking of the algorithms in the related experiments and discussions. This work does not pursue obtaining a final winner algorithm from comparisons but to discover and discuss the fundamental aspect of CE from viewpoint of chaotic optimization and optimization mechanism of CE algorithm with different chaotic systems behind the evaluation results. Some study subjects are analysed and discussed taking into account evaluation results and statistical tests. These topics are the relationship between distribution characteristic of chaotic system and optimization performance, algorithm ranking, interactive CE algorithm, disadvantages and improvement approaches of CE algorithm, a notation system of CE, and so forth. These research subjects present the originality and primary contribution of this paper.

Following this introductory section, an overview of the CE algorithm framework and chaotic systems used in this paper are reported in Section 2. We present several new CE algorithms in this paper, which demonstrates scalability of the chaotic algorithm framework. Interactive CE (ICE) algorithm framework is introduced in Section 2 as well. In Section 3, experimental evaluations of chaotic evolution are performed and some statistical tests are applied. We discuss our proposed CE algorithms and obtained evaluation results. In Section 4, we analyse our proposed new IEC algorithm. We discuss some issues based on ICE experimental simulation using a pseudo-IEC user, which is implemented by a Gaussian mixture model. The topic on algorithmic notation of CE is defined for its further development and study. Finally, we conclude the whole work, and some open topics, further opportunities, and future works are discussed in Section 5.

## 2. Chaotic Evolution Algorithm Framework, Interactive Chaotic Evolution, and Chaotic System

### 2.1. A Brief Review of Chaotic Evolution Algorithm

Deterministic system and stochastic system are two views in which we understand and describe the nature in philosophy and in science. They lead to two corresponding algorithm methodologies in optimization field, that is, deterministic and stochastic optimization algorithms. Evolutionary computation can be partially categorized into the later one, that is, stochastic optimization algorithm. However, since chaotic phenomenon and mechanism were found [3, 4], chaos and chaotic system are as well a tool to describe and study the nature besides deterministic and stochastic systems. In optimization field, there must be a set of chaotic optimization algorithms inspired and normalized by chaotic philosophy and methodology. This is the fundamental motivation that prompts us to discover and study new optimization algorithms in the viewpoint of chaotic optimization.

The concepts of “evolution” and “chaos” have more the same characteristics in common [6]. First, both words refer to the phenomena that need to be explained and to the theories that are supported to do the explaining [10]. Second, there must be an iteration in both evolution and chaos processes, so the phenomena of them can appear. Third, the concepts and theories of evolution and chaos influence and enhance all research works by introducing and fusing the fundamentals of their concepts and approaches.

Inspired from chaotic motion of nonlinear system that has an ergodicity property, a chaotic ergodicity based EC algorithm,* chaotic evolution*, was proposed and studied recently [6]. It simulates the chaotic motion in a search space for implementing optimization. Because chaotic motion has an ergodicity property, the proposed algorithm can guarantee its global convergence partially. The scalability of the algorithm is better than other EC algorithms by introducing different chaotic systems.

**Figure 1:**
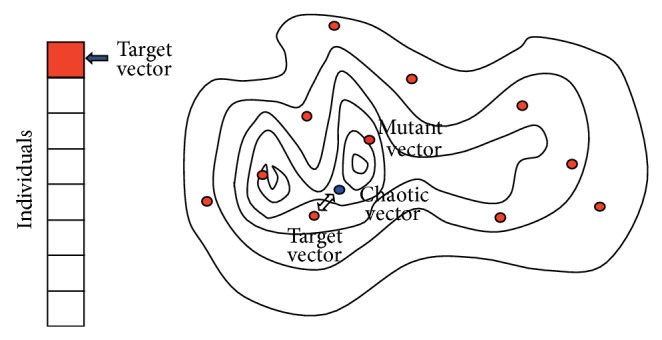
Chaotic evolution algorithm framework; there are three special vectors in chaotic evolution algorithm, a target vector that is an individual in the population, a mutant vector that simulates the chaotic motion from a chaotic system, and a chaotic vector that is made by conducting crossover operation on a target vector and a mutation vector.

Suppose that an array on the left side of Figure 1 presents individuals, contour lines at the right side are a fitness landscape, and circles on the landscape are the individuals. CE algorithm for one search generation is described below. It is repeated until a satisfied solution is found or the search reaches to the desired generations.

(1) Choose one individual as a target vector.
(2) Obtain a chaotic parameter from a chaotic system
  (1) $$\begin{array}{c} \text{C}{\text{P}}_{i}=\text{Chaotic}\;\text{System}\;\left(\text{C}{\text{P}}_{i-1}\right). \end{array}$$
(3) Make a mutant vector by
  (2) $$\begin{array}{c} \text{Mutant}\;{\mathrm{Vec}.}_{i}=\text{Target}\;{\mathrm{Vec}.}_{i}*\left(1+D_{i}*\text{C}{\text{P}}_{i}\right). \end{array}$$
(4) Generate a chaotic vector by crossing the target vector and the mutant vector
  (3) $$\begin{array}{c} \text{Chaotic}\;{\mathrm{Vec}.}_{i}=\text{Cr}\left(\text{Mutant}\;{\mathrm{Vec}.}_{i},\text{Target}\;{\mathrm{Vec}.}_{i}\right). \end{array}$$
(5) Compare the target vector and the chaotic vector and choose whichever a better one as offspring in the next generation.
(6) Go to (1) and generate other offspring until all individuals are replaced with offspring in the next generation.

The terms of* vector* and* individual* mean the same search points. (1)–(4) are summarized as in (1), (2), and (3). This sketches the chaotic evolution algorithm which is easily implemented, where *D*_*i*_ is called a direction factor and CP_*i*_ is a chaotic parameter. There are several chaotic evolution variations in (1) for selecting different chaotic systems, crossover methods in (4), and others. After initial population needs fitness evaluation once, only the chaotic vector in (5) needs a fitness evaluation in every generation.

### 2.2. Interactive Chaotic Evolution Algorithm

Interactive evolutionary computation (IEC) is a niche research field in EC community [11]. The primary objective of IEC purposes solving the optimization problems that should embed subjective evaluation of real human into the optimized solution. Most of the evaluation spaces of IEC application are hard to be explicitly expressed or cannot be represented at all, so the real human's evaluation is necessary to achieve the final optimal solution. However, human fatigue is a serious issue for an IEC optimization application, because a real human has his or her evaluation limitation, and physiological or psychological tolerance capacity comparing with a tirelessness computer. So to relieve human's fatigue is a practical problem in IEC algorithm study and application.

From a framework viewpoint, there are three main parts in an IEC based optimization system. They are (1) a target system that is optimized, (2) an IEC algorithm (including an IEC interface) that conducts actual optimization function, and (3) a real human who provides his or her evaluation. The target system and the real human are fixed in an IEC optimization application, so there is a study subjective to relieve human's fatigue in the IEC algorithm part. One is to enhance optimization performance of IEC algorithm. The other is to improve IEC interface for a user friendly interaction.

If we implement a CE algorithm in an IEC application and replace the fitness function with a real human's evaluation, the IEC framework is an implementation of interactive chaotic evolution (ICE). In the nature of ICE algorithm, there is a paired comparison-based mechanism when surviving an offspring between chaotic vector and target vector. In an IEC application, these two vectors present two IEC solutions for a real human to perform evaluations. Comparing with other IEC algorithms, such as interactive genetic algorithm (IGA), this paired comparison mechanism benefits real human's evaluation rather than evaluating all individuals together as in IGA. If the ICE algorithm can present significantly optimization performance compared to other paired comparison-based IEC algorithms, such as interactive differential evolution (IDE) [12] or its variations [13], ICE can be a practical algorithm implementation in IEC framework and enrich IEC algorithm family. We will investigate this subject in this paper.

### 2.3. Chaotic Systems

The search function of CE is implemented by generating a chaotic vector. It is decided by chaotic parameter from a chaotic system, which has the ergodicity property. By applying a variety of chaotic systems in the CE algorithm framework, we can implement its variations in different way. In this study, we introduce four chaotic systems, that is, logistic map, tent map, Gaussian map, and Hénon map to investigate the optimization performances of CE with these four chaotic maps. Here, we make a brief review on these chaotic maps.

#### 2.3.1. Logistic Map

**Figure 2:**
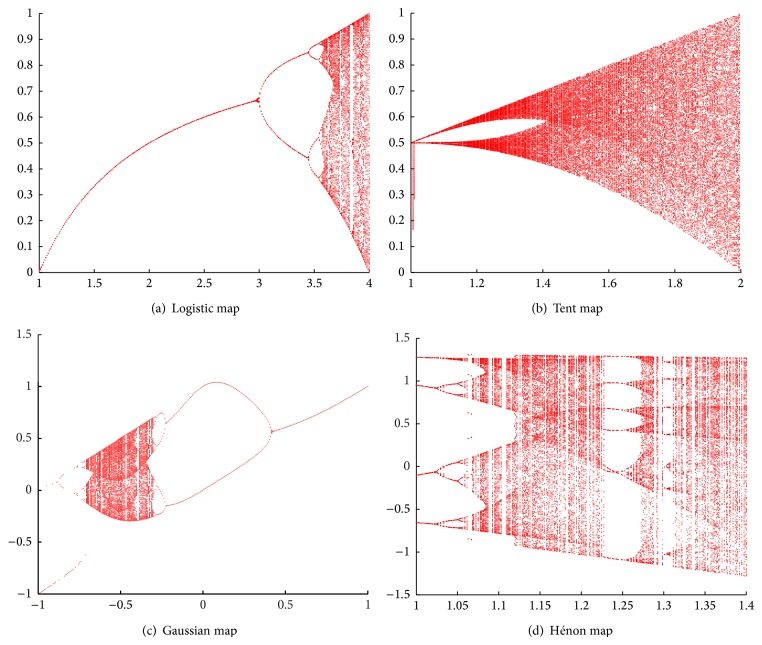
Bifurcation digrams of (a) logistic map, (b) tent map, (c) Gaussian map, and (d) Hénon map. Parameter *μ* in logistic map can be set at (0,4]; chaos happens when *μ* = 4 in (a). Parameter *μ* in tent map can be set at [1,2]; chaos happens when *μ* = 2 in (b). Parameter *β* in tent map can be set at [−1, +1]; chaos happens when *α* = 6.20 and *β* = −0.5 in (c). Parameters *a* = 1.4 and *b* = 0.3 are a classic setting in Hénon map; one of its invariant points on the attractor, $x=(\sqrt{609}-7)/28=0.631354477$ and $y=\left(3\sqrt{609}-7\right)/280=0.189406343$, can be used as an initial value setting.

The logistic map is a polynomial mapping with two degrees that can arise from a chaotic phenomenon by a simple nonlinear system easily [14]. Equation (4) shows its mathematical expression, where *μ* is a parameter that decides the behaviour of system; *μ* is usually set at (0,4]. For the values of *μ* > 4, the map does not project interval [0,1] into itself. When *μ* = 4, the system arises from chaotic phenomenon. The bifurcation diagram of logistic map is shown in Figure 2(a) that presents the whole system behaviour:

(4) $$\begin{array}{c} x_{n}=\mu *x_{n-1}*\left(1-x_{n-1}\right). \end{array}$$

#### 2.3.2. Tent Map

The tent map is defined as in (5) whose function curve is like the tent-like shape. The parameter *μ* is within (0,2]. Depending on the value of *μ*, the tent map demonstrates a range of dynamical behaviours from predictable to chaotic (see its bifurcation diagram in Figure 2(b)). When *μ* equals 2, the map becomes chaotic:

(5) $$\begin{array}{c} x_{n}=\left\{\begin{array}{cc} \mu *x_{n-1} & \text{if}\;0\leq x_{n}<0.5,\\ \mu *\left(1-x_{n-1}\right) & \text{if}\;\;0.5\leq x_{n}\leq 1. \end{array}\right. \end{array}$$

#### 2.3.3. Gaussian Map

The Gaussian map is a nonlinear map that is given by a Gaussian function (equation (6)), whose bifurcation diagram resembles a mouse (Figure 2(c)). The system can become chaotic, when the parameters *α* and *β* are set at 6.20 and −0.5, respectively:

(6) $$\begin{array}{c} x_{n}=\exp\left(-\alpha *x_{n-1}^{2}\right)+\beta . \end{array}$$

#### 2.3.4. Hénon Map

The Hénon map has two points (*x*_*n*_, *y*_*n*_) in the plane, and it maps them into a new point (equation (7)). The system behaviour depends on its parameters, that is, *a* and *b*. Different from the other chaotic maps, Hénon map is a two-dimensional nonlinear system. It is chaotic when its parameters have values of *a* = 1.4 and *b* = 0.3, which is a classic parameter setting. For other values of *a* and *b*, the map may be chaotic or intermittent or converge to a periodic orbit (Figure 2(d)). The Hénon map does not have a strange attractor for all values of the parameters *a* and *b*, but there are invariant points on the attractor; $x=(\sqrt{609}-7)/28=0.631354477$ and $y=(3\sqrt{609}-7)/280=0.189406343$ are examples of them:

(7) $$\begin{array}{c} x_{n}=y_{n}+1-a*x_{n}^{2},\\ y_{n}=b*x_{n}. \end{array}$$

## 3. Chaotic Evolution Optimization Evaluation Analysis and Discussion

### 3.1. Experimental Settings and Results

We use 25 benchmark functions from [15] to evaluate our proposed CE algorithms with new introduced chaotic systems.  Table 1 presents their type, characteristic, search bound, and optimum fitness value of these benchmark functions. For all benchmark functions, its dimensional settings are 10 and 30. For all algorithms, it runs up to 1000 generations with 30 trails running. The population size is 5∗dimension; that is, 50 and 150 are for 10D and 30D benchmark functions, respectively.

There are five CE algorithms in our evaluation, that is, CE-logistic, CE-tent, CE-Gauss, CE-Hénon-rand, and CE-Hénon-attrac. Two DE algorithms (DE/best/1/bin and DE/rand/1/bin) are used to be compared with CE algorithms [16]. Abbreviations and their meaning are in Table 2. CE with Hénon map has two initialization methods. One uses random value within (0, 1] (CE-Hénon-rand), and the other uses an attractor, $x=(\sqrt{609}-7)/28=0.631354477$ and $y=(3\sqrt{609}-7)/280=0.189406343$ (CE-Hénon-attrac.). Other chaotic systems of chaotic evolution algorithms use the uniform random number within (0, 1] as their initial value. The evaluation hardware environment is PC with Windows 8.1 (x64) on Intel(R) Core(T) i7-4500 (CPU@1.80 GHz, 2.39 GHz, 4 GRAM); the algorithms are implemented in MATLAB (R2011b).

We apply Wilcoxon sign-ranked test and Friedman test on the fitness value at 1000th generation and make two groups with and without DE algorithms to rank the algorithms. We use Bonferroni-Dunn's test to check the significance of algorithm rank in both groups. Tables 3 and 4 are the fitness mean value of ALL algorithms at 1000th generation for 10D and 30D benchmark functions, respectively.

### 3.2. Chaotic Evolution Optimization Performance

One of the objectives in this paper is to investigate the optimization performance by using different chaotic systems in CE algorithm framework. Tables 3 and 4 show the mean of fitness value at the 1000th generation of each algorithm for 10D and 30D benchmark functions. The fitness values with bold font and italic font indicate the best and the worst fitness values among five proposed CE algorithms. From these two tables, we can conclude that CE with tent map obtains the worst fitness value for most of 10D and 30D benchmark functions. CE with Gaussian map and Hénon map using attractor initialization obtains the best results in 10D and 30D benchmark functions. It presents the fact that CE algorithm with tent map is less useful for most of benchmark tasks practically.

We apply Wilcoxon sign-ranked test (*P* < 0.05) on results with the best and the worst fitness values; most of the results indicate that there is a significant difference between all pairs. It addresses the fact that the optimization performance of CE is influenced significantly by the chaotic system.

Figure 2 shows the output ranges of four bifurcation diagrams of chaotic system used in our proposed CE algorithms. For further investigating the output distribution of each chaotic system, we calculate their output distribution in equal intervals and their percentages in Table 5.

There are some relationships between output distribution characteristic of chaotic system and optimization performance of CE. In logistic map, most of the system outputs cover the intervals of (0,0.1] and (0.9,1], whose percentages are up to 20.55% and 20.52%, respectively. When making a mutation vector from a target vector, the mutation vector will locate in the position near the target vector with adding the length of (0,0.1]∗target and far from it with adding that of (0.9,1]∗target. It presents exploitation and exploration functions of CE algorithm with the logistic map. Most of the output values locate in the interval (0,0.1] in tent map, which indicates that the exploitation capability of CE with tent map is better than the others. However, its evolution speed is slow due to lack of exploration function. This is a primary reason why CE with the tent map has the worst optimization results in most of benchmark tasks. It is a disadvantage of CE-tent algorithm. The outputs of Gaussian map and Hénon map seem averagely to locate in the intervals among (0,0.5] and (0,1], respectively. These distributions ensure the exploitation and exploration functions of CE with Gaussian map and Hénon map. So they show the better optimization performance among the five CE algorithms averagely.

### 3.3. Comparison with Differential Evolution

We select two DE algorithms (DE/best/1/bin and DE/rand/1/bin) as two competitors to make a comparative evaluation with our proposed five chaotic evolution algorithms. The † and ‡ marks in Tables 3 and 4 show that our proposed CE algorithms have significantly better or equal performance to the evaluation performances of DE/best/1/bin and DE/rand/1/bin from the Wilcoxon sign-ranked tests (*P* < 0.05), respectively. By comparing the winner number of CE algorithms in Tables 3 and 4, CE algorithms, which are applied to higher dimensional benchmark functions (30D), can obtain better optimization performance than that applied to lower dimensional benchmark functions (10D). Particularly for the benchmark tasks, F11, F13, F14, F18, F19, F20, and F25, whose fitness landscapes are more complex, our proposed algorithms can obtain significantly better optimization performance.

### 3.4. Algorithm Ranking

We apply Friedman test on our proposed five CE algorithms and two DE algorithms to make an algorithm rank.  Table 6 is the rank results of seven competitive algorithms. We can observe that DE/best/1/bin is the winner algorithm for both 10D and 30D benchmark functions averagely. However, for the F10–F14, F18–F20, its ranks become down because optimization results of our proposed algorithms are better than those in these benchmark tasks. Proposed CE algorithms have powerful optimization capability in the benchmark tasks with more complex fitness landscape from Table 6, especially for the higher dimensional tasks (30D). From evaluation metric of the average rank, the proposed CE algorithms have almost the same rank for some pairs, for example, CE-logistic and CE- Hénon-rand and CE-Gauss and CE-Hénon-attrac. in the 10D group. The same observations can be found in the 30D group as well.  Table 7 is the algorithm rank only among five CE algorithms. CE-Gauss and CE-CE-Hénon-attrac. are the winner algorithm from the metric of average rank. The rank scores among five algorithms are not different so much. We suppose that the comparison within five CE algorithms and the comparison within CE and DE (seven algorithms) may not present significant difference when they are applied to a variety of benchmark tasks, that is, in a large sample condition.

We apply Bonferroni-Dunn's tests on the results of Friedman test to evaluate our hypothesis whether these algorithms have a significant difference. The tests are applied on a group with DE to compare CE and DE and on a group without DE to compare the CE algorithms with different chaotic systems. Both tests are for the 10D and 30D benchmark tasks. The evaluation metrics of critical difference (CD) are calculated with (8), in which *k* is the number of algorithms and *N* is the number of benchmark functions:

(8) $$\begin{array}{c} \text{CD}=q*\sqrt{\frac{k*\left(k+1\right)}{6*N}}. \end{array}$$

In our evaluation, *k* = 7 and *k* = 5 are for the groups with and without DE and *N* = 25 for both groups. Parameter *q* is critical value for nonparametric multiple-comparison testing with a control [17]; we use *q*_*α*=0.01,*k*=7_ = 3.144 and *q*_*α*=0.05,*k*=7_ = 2.639 and *q*_*α*=0.01,*k*=5_ = 3.024 and *q*_*α*=0.05,*k*=5_ = 2.498 in our calculations of CD. The results are CD_*q*_*α*=0.01,*k*=7__ = 1.92 and CD_*q*_*α*=0.05,*k*=7__ = 1.61 and CD_*q*_*α*=0.01,*k*=5__ = 1.35 and CD_*q*_*α*=0.05,*k*=5__ = 1.11. By comparing with the average values of mean ranking, there are not any CD values excluding average values in each group (taking the algorithm with the less rank value as a control algorithm). These results indicate that (1) CE algorithms have the same optimization capabilities comparing with the DE algorithms, and (2) there is not any significant difference among CE algorithms with different chaotic systems setting, when CE is applied to a variety of benchmark tasks (in the viewpoint of large sample condition).

## 4. Interactive Chaotic Evolution Optimization Evaluation Analysis and Discussion

### 4.1. Experimental Settings and Results

**Figure 3:**
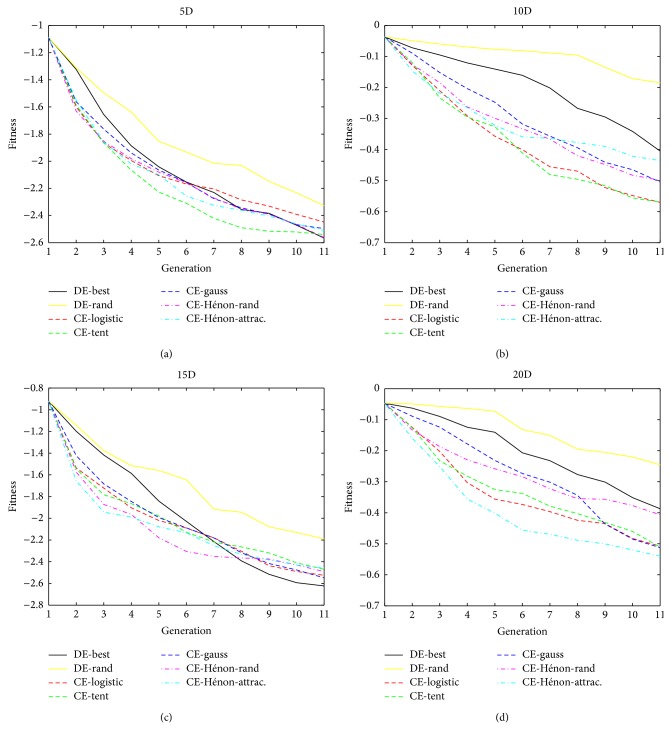
Interactive chaotic evolution convergent curves of 5D, 10D, 15D, and 20D Gaussian mixture model. All the ICE algorithms present fast convergent speed, especially in 10D and 20D tasks. IDE/best can obtain better final results than ICE, but it needs more fitness evaluation (i.e., more human subjective evaluation in IEC application) to find the best base vector. From the metric of fitness evaluation time, ICE is significantly better than IDE/best when these two algorithms call the same fitness function time.

We propose using CE algorithm framework to implement a new IEC algorithm, interactive chaotic evolution (ICE). It is one of originalities and contributions in this paper. We use a Gaussian mixture model (equation (9)) as a pseudouser to evaluate ICE algorithm. Dimension setting of the Gaussian mixture model is 3, 5, 7, 10, 13, 15, 17, and 20. The population size of the algorithms is 20 and we evaluate them with 20 generations to simulate the characteristic of IEC algorithms, that is, with less population size and less generation. Other parameter settings are as well in Section 3; we use interactive version of DE, that is, IDE, to compare it with the new proposed ICE.  Table 8 is the mean value of all the algorithms at 20th generation; we apply Wilcoxon sign-ranked test to evaluate the significance of results.  Figure 3 shows convergent curves of 5D, 10D, 15D, and 20D Gaussian mixture models:

(9) $$\begin{array}{c} \;\;\text{GMM}\left(x\right)=\overset{k}{\underset{i=0}{\sum }}a_{i}\exp\left(-\overset{n}{\underset{j=0}{\sum }}\frac{{\left(x_{ij}-\mu_{ij}\right)}^{2}}{2\sigma_{ij}^{2}}\right), \end{array}$$

where

(10) $$\begin{array}{c} \sigma =\left(\begin{array}{cccccccccc} 1.5 & 1.5 & 1.5 & 1.5 & 1.5 & 1.5 & 1.5 & 1.5 & 1.5 & 1.5\\ 2 & 2 & 2 & 2 & 2 & 2 & 2 & 2 & 2 & 2\\ 1 & 1 & 1 & 1 & 1 & 1 & 1 & 1 & 1 & 1\\ 2 & 2 & 2 & 2 & 2 & 2 & 2 & 2 & 2 & 2 \end{array}\right),\\ \mu =\left(\begin{array}{cccccccccc} -1 & 1.5 & -2 & 2.5 & -1 & 1.5 & -2 & 2.5 & -1 & 1.5\\ 0 & -2 & 3 & 1 & 0 & -2 & 3 & 1 & 0 & -2\\ -2.5 & -2 & 1.5 & 3.5 & -2.5 & -2 & 1.5 & 3.5 & -2.5 & -2\\ -2 & 1 & -1 & 3 & -2 & 1 & -1 & 3 & -2 & 1 \end{array}\right),\\ \;\;a_{i}={\left(\begin{array}{cccc} 3.1, & 3.4, & 4.1, & 3.0 \end{array}\right)}^{T}. \end{array}$$

### 4.2. Interactive Chaotic Evolution and Its Paired Comparison Mechanism

We analyse the optimization results of CE algorithms at the 1000th generation in Section 3. From the evaluation results and statistical tests, our proposed CE algorithms have better optimization performance in the initial 10 or 20 generations than that of DE algorithms in some benchmark tasks. This feature of CE algorithm can benefit the IEC [11], so ICE algorithm should be a powerful algorithm to the IEC application.

One of the characteristics in IEC is that it is required to run with less generation and less population size to solve the user fatigue problem. IEC user compares each individual with others kept in their memory and the mental load and fatigue increase. Reference [18] pointed out that human cannot process more than five to nine different items simultaneously. The population sizes of many IEC applications frequently exceed this memory limitation, and displaying more than five to nine sounds or movies, that is, time series object, to an IEC user, is not practical. Paired and multiple comparison-based IEC solves this problem by replacing the comparison of all individuals with paired or multiple comparisons [13]. It is therefore expected to reduce IEC user fatigue.

In the running process of CE algorithm framework (Section 2.1), there are paired comparison mechanisms in its principle when surviving the target vector and the chaotic vector into the next generation. If we apply the ICE algorithm for a concrete IEC application, it is expected that the optimization performance of ICE may be better than that of IDE from the initial optimization results from Section 3.  Table 8 sketches that ICE algorithms are significantly better than or are as the same as IDE/best/1/bin and IDE/rand/1/bin. In the viewpoint of fitness evaluation number, ICE algorithms are as the same as IDE/rand/1/bin but less than IDE/best/1/bin, because human user must compare all individuals together to select the best individual as the base vector. ICE presents better optimization performance than that of IDE/best/1/bin. That is, it can reduce human evaluation time and obtain optimization results better or at least as the same as IDE/best/1/bin algorithms, especially in initial generation shown in Figure 3.

### 4.3. Fusion of Chaotic Evolution and Differential Evolution

Evaluation results indicate that the convergent speeds of CE algorithms become down in some benchmark functions, especially in 15D tasks (Figure 3). However, convergence speeds of DE algorithms seem to be with the same tendency in Figure 3. If we fuse CE and DE together to make a hybrid algorithm optimization framework, the performance of hybrid algorithm should expectedly be better than any of the single algorithms. For example, when the convergence speeds of DE become slow, we can apply the CE algorithm with multiple chaotic systems to increase its population diversity and vice versa. Fusion algorithm can be implemented in dimension level, individual level, and generation level. This is a promising topic for the further investigation.

There are multiple implementations of the CE algorithm framework. We initially have investigated their optimization performance and output distribution of chaotic systems. Another opportunity for enhancing optimization performance of CE is to fuse multiple chaotic systems in one CE algorithm. It is as well a promising research topic for further study. There are three methods to implement this algorithm framework. First is to apply one chaotic system for a certain generation and change another for the following generations by observing the algorithm convergent speed or other evaluation metrics. Second is to fuse CE with multiple chaotic systems in individual level; that is, some of individuals are searching by the law of one chaotic system and some of the others by that of other chaotic systems. Third is to apply different chaotic systems on different dimensions of one individual. The landscape or search situation is different from all dimensions, and this must be matched by different dimensional searches with different chaotic systems. We can design a CE algorithm with multiple chaotic systems by obtaining the fitness landscape information in the whole or the particle dimensions. Some methods of analysing and approximating the fitness landscape information can be found in [19, 20]. This is a research subject in our future work.

### 4.4. Algorithmic Notation of Chaotic Evolution

We develop an algorithmic notation system for a better explanation and further development of CE algorithms. The actual search function of chaotic algorithms is implemented by a chaotic system, in which there are some parameters. This is one element in chaotic evolution. Another parameter of chaotic evolution is the crossover rate.

We abbreviate chaotic evolution as CE and use the notation format CE/*x*/*y*/*z* to make a note of a certain chaotic evolution algorithm, where *x*, *y*, and *z* present a chaotic system, the parameters of chaotic system, and a crossover rate, respectively. For example, the CE algorithm with Gaussian map in this paper can be a notation as “CE/Gaussian/(*α* = 6.2, *β* = −0.5, random initialization)/1.”

## 5. Conclusion and Future Works

In this paper, we develop a chaotic optimization algorithm that is theoretically supported by the fundamental of chaos theory. We introduce four chaotic systems in a well-designed CE algorithm framework to implement several CE algorithms, that is, CE-logistic, CE-tent, CE-Gauss, CE-Hénon-rand, and CE-Hénon-attrac. We analyse the optimization performances of these developed algorithms. A comparative evaluation is conducted by applying the Wilcoxon sign-ranked test and the Friedman test with two DE algorithms. We propose a new IEC algorithm, that is, ICE that has a paired comparison mechanism in its search scheme. A series of topics on optimization performances of chaotic evolution algorithms, comparison with DE algorithms, algorithms rank, interactive chaotic evolution, and fusion of these algorithms for enhancing performance are analysed and discussed.

In this paper, we do not pursue obtaining the results of the best winner algorithm but analyse and discuss the algorithm optimization mechanism of CE and the philosophy behind it. In the modern scientific world, there are two primary philosophies and methodologies to describe and study nature world from the determinism and probability viewpoint. In the optimization field, there are corresponding deterministic and stochastic optimization algorithms that are supported by these two methodologies. EC is one of stochastic optimization algorithms. However, after the discoveries of chaotic phenomena and systems, chaos becomes the third methodology to study the nature world. Chaotic optimization algorithm should therefore be researched and developed in optimization field. Chaotic evolution is one of the implementations, although it has many disadvantages in its search scheme, such as searching without considering fitness landscape, exploration, and exploitation depending on chaotic system rather than adaptive mechanism. In the theoretical analysis of CE, we will analyse concrete CE algorithm with Markov chain [6] and other mathematical models. They are interesting research topics on evaluating CE performance by a variety of benchmark functions and comparing it with other metaheuristics algorithms [7–9, 21]. We will further investigate these topics in our future work and hopefully discover new knowledge behind CE algorithm. It should benefit both chaos theory and evolutionary optimization hopefully.

## Figures and Tables

**Table 1:** Test functions (Uni = unimodal, Multi = multimodal, Sh = shifted, Rt = rotated, GB = global on Bounds, HC = hybrid composition, and NM = number matrix).

Number	Type	Characteristic	Bounds	Optimum fitness
*f*_1_	Uni	Sh sphere	[−100,100]	−450
*f*_2_		Sh Schwefel 1.2		−450
*f*_3_		Sh Rt Elliptic		−450
*f*_4_		*f*_2_ with noise		−450
*f*_5_		Schwefel 2.6 GB		−310

*f*_6_	Multi	Sh Rosenbrock	[−100,100]	390
*f*_7_		Sh Rt Griewank	[0,600]	−180
*f*_8_		Sh Rt Ackley GB	[−32,32]	−140
*f*_9_		Sh Rastrigin	[−5,5]	−330
*f*_10_		Sh Rt Rastrigin	[−5,5]	−330
*f*_11_		Sh Rt Weierstrass	[−0.5,0.5]	90
*f*_12_		Schwefel 2.13	[*π*, *π*]	−460
*f*_13_		Sh expanded F8F2	[−3,1]	−130
*f*_14_		Sh Rt Scaffer F6	[−100,100]	−300

*f*_15_	Hybrid	HC function	[−5,5]	120
*f*_16_		Rt HC function 1		120
*f*_17_		*f*_16_ with noise		120
*f*_18_		Rt HC function 2		10
*f*_19_		*f*_18_ with basin		10
*f*_20_		*f*_18_ with GB		10
*f*_21_		Rt HC function 3		360
*f*_22_		*f*_21_ with NM		360
*f*_23_		NC Rt *f*_21_		360
*f*_24_		Rt HC function 4		260
*f*_25_		*f*_24_ without bounds		260

**Table 2:** Algorithm parameter setting and abbreviations of the algorithms used in evaluation.

Abbreviation	Meaning
CE-logistic	Chaotic evolution with logistic map (random initialization within (0, 1], *μ* = 4).
CE-tent	Chaotic evolution with tent map (random initialization within (0, 1], *μ* = 2).
CE-Gauss	Chaotic evolution with Gaussian map (random initialization within (0, 1], *α* = 6.2, *β* = −0.5).
CE-Hénon-rand	Chaotic evolution with Hénon map (random initialization within (0, 1], *a* = 1.4, *b* = 0.3).
CE-Hénon-attrac.	Chaotic evolution with Hénon map (initialization with attractors explained in Figure 2, *a* = 1.4, *b* = 0.3).
CE crossover rate	1

DE/best/1/bin	DE with the best individual as base vector.
DE/rand/1/bin	DE with random individual as base vector.
DE scale factor *F*	1
DE crossover rate	1

**Table 3:** Mean fitness values of F1–F25 with 10D. The fitness values in bold and italic font are the best and worst optimization results among the five chaotic evolution algorithms, respectively. The (†) and (‡) marks mean that they are significantly better than or as the same as DE/best/1/bin and DE/rand/1/bin, respectively, by Wilcoxon sign-ranked test (*P* < 0.05).

Function	DE-best	DE-rand	CE-logistic	CE-tent	CE-Gauss	CE-Hénon-rand	CE-Hénon-attrac.
F1	−449.998	−414.47	2998.806	*5171.277 *	1925.366	1768.834	**1661.428**
F2	−440.516	−421.736	4219.061	*7585.492 *	2667.176	3855.883	3568.253
F3	1583.076	215058.7	3624018	26036921	2686083	4846787	*3973677 *
F4	−283.931	−402.19	6487.652	*8960.625 *	4226.79	4723.953	5358.734
F5	−310	−309.972	3775.085	*9448.24 *	3986.86	4148.611	1892.268
F6	446.6181	35496.74	287017050.30	628335744.87	272134693.96	*78198866 *	22561790
F7	−177.747	−177.865	−97.4097	−*22.6933 *	−127.847	−105.609	−121.71
F8	−119.992	−119.893	−119.635	−*119.38 *	−119.62	−119.604	−119.644
F9	−308.575	−306.239	−284.906	−*269.758 *	−282.345	−284.484	−282.21
F10	−291.876	−282.316	−254.408	−*232.193 *	−261.758	−258.089	−264.328
F11	98.34748	94.5809	98.25204^†^	*99.94741 *	97.20956^†^	99.0253^†^	97.52258
F12	321.4523	4968.164	20706.57	53091.15	33802.12	28609.12	16818.88
F13	−126.975	−126.213	−125.596	−*123.459 *	−126.954^†‡^	−124.246	−126.658^†‡^
F14	−296.159	−296.567	−296.327^†^	−*295.874 *	−296.44^†‡^	−296.269^†^	−296.384^†‡^
F15	476.4005	557.9363	573.2322^‡^	*746.0844 *	612.5314^‡^	621.6522^‡^	547.8405^‡^
F16	302.9829	337.3688	378.9592	*452.6815 *	377.3656	391.311	356.5135
F17	323.7027	326.4775	409.4423	*474.5775 *	401.6362	426.4379	381.8373
F18	869.8405	578.9428	910	910	910	*910.1011 *	910
F19	869.4931	591.2802	910	910	905.6865	*910.5839 *	910
F20	866.7625	610.5591	910	910	905.6865	*910.5858 *	910
F21	1079.758	929.7804	1623.005	*1691.75 *	1545.881	1657.526	1574.536
F22	1222.383	1164.546	1320.278	*1406.045 *	1307.327	1309.518	1298.61
F23	1066.17	970.7627	1647.835	*1701.935 *	1539.408	1649.8	1563.626
F24	824.7887	494.8996	1450.171	*1498.591 *	1376.52	1398.889	1270.075
F25	1989.595	2001.341	2010.443	2009.772	2018.292	*2021.227 *	2014.102

**Table 4:** Mean fitness values of F1–F25 with 30D. The explanations of special marks are as the same as in Table 3.

Function	DE-best	DE-rand	CE-logistic	CE-tent	CE-Gauss	CE-Hénon-rand	CE-Hénon-attrac.
F1	97.24339	10626.17	33437.54	*44676.41 *	22489.18	34997.74	27669.15
F2	1513.336	22808.96	28931.33	*38786.76 *	23073.34^‡^	31553.89	26444.3
F3	3203063	91581615	155259859.97	*435879301.22 *	101212084.10	212799570.04	139163745.08
F4	10475.36	31198.64	32279.57^‡^	*43413.27 *	28657.47^‡^	37707.88	33388.45
F5	3753.477	12014.48	18617.58	*27065.35 *	19258.04	21922.04	18336.66
F6	10041703	917911267.58	11016735211.66	*18010453533.81 *	6390791853.98	11349392992.19	6359560226.21
F7	−165.651	98.12331	1042.799	*1718.531 *	803.677	1145.698	996.1877
F8	−119.769	−119.03	−119.027^‡^	−*118.906 *	−119.022^‡^	−119.041^‡^	−119.031^‡^
F9	−224.472	−80.3587	−4.98262	*17.25496 *	−26.9584	−15.6137	−20.0503
F10	−157.64	−16.854	170.6209	*238.9339 *	156.3653	133.2562	132.4319
F11	124.2699	129.963	125.7166^†‡^	*130.6959* ^‡^	123.5876^†‡^	130.007^‡^	124.5107^†‡^
F12	39573.98	945070.8	722534^‡^	*1120996 *	1097621	875280.5^‡^	678438.7^‡^
F13	−94.4806	−72.5022	−78.4622^‡^	−64.6016	−102.475^†‡^	−*58.4634 *	−94.5819^†^
F14	−286.7	−286.454	−286.813^†‡^	−*286.369* ^‡^	−286.822^†‡^	−286.57^†‡^	−286.962^†‡^
F15	712.6463	679.5186	907.8008	*1046.292 *	832.5582	943.7358	899.282
F16	394.5135	445.3347	645.2657	*811.5967 *	633.273	684.447	610.1459
F17	456.3692	489.36	698.1601	*882.8512 *	725.3444	715.6857	664.8518
F18	924.3655	976.0293	910^†‡^	910^†‡^	910^†‡^	*1029.592 *	910^†‡^
F19	927.3029	973.5324	910^†‡^	910^†‡^	910^†‡^	*1026.967 *	910^†‡^
F20	927.4663	971.5176	910^†‡^	910^†‡^	910^†‡^	*1014.319 *	910^†‡^
F21	1335.783	1319.538	1665.937	*1697.951 *	1638.145	1657.629	1657.872
F22	1260.922	1312.237	1552.663	*1698.287 *	1545.124	1569.841	1559.818
F23	1418.319	1374.563	1667.109	*1695.831 *	1633.517	1659.605	1662.561
F24	1218.33	1210.63	1618.306	*1638.226 *	1614.015	1593.555	1621.32
F25	1890.716	1944.179	1891.82^†‡^	1889.155^†‡^	1912.471^‡^	*1951.921 *	1891.442^†‡^

**Table 5:** System output total number and its percentage in each interval of logistic map, tent map, Gaussian map, and Hénon map with attractor initiation after 10^5^ iterations. The output of Hénon map with random initiation is out of the interval (0,1].

Interval	Logistic		Tent		Gaussian		Hénon-attrac.	
(0,0.1]	20552	20.55%	99952	99.95%	7319	10.77%	2193	4.52%
(0.1,0.2]	9051	9.05%	6	0.01%	13830	20.36%	3272	6.74%
(0.2,0.3]	7396	7.40%	9	0.01%	10366	15.26%	3263	6.72%
(0.3,0.4]	6674	6.67%	3	0.00%	13236	19.48%	7039	14.49%
(0.4,0.5]	6353	6.35%	7	0.01%	23191	34.13%	5410	11.14%
(0.5,0.6]	6473	6.47%	6	0.01%	0	0.00%	5505	11.34%
(0.6,0.7]	6691	6.69%	1	0.00%	0	0.00%	7601	15.65%
(0.7,0.8]	7296	7.30%	3	0.00%	0	0.00%	5606	11.54%
(0.8,0.9]	8996	9.00%	6	0.01%	0	0.00%	3402	7.01%
(0.9,1]	20518	20.52%	7	0.01%	0	0.00%	5272	10.86%

**Table 6:** Algorithms' rank with DE by Friedman test for F1–F25 of 10D and 30D, respectively. The abbreviation meanings are in Table 2.

Function	DE-best	DE-rand	Logistic	Tent	Gauss	Hénon-rand	Hénon-attrac.
	10-dimensional function						
F1	1.00	2.00	5.07	6.93	4.33	4.47	4.20
F2	1.03	1.97	5.17	6.87	3.73	4.73	4.50
F3	1.03	1.97	4.40	6.90	3.77	5.33	4.60
F4	1.70	1.30	5.30	6.60	4.03	4.50	4.57
F5	1.00	2.00	4.63	6.83	4.83	5.00	3.70
F6	1.00	2.00	5.20	6.77	4.50	5.03	3.50
F7	1.33	1.67	5.17	6.77	3.83	5.03	4.20
F8	1.07	2.23	4.30	6.70	4.43	4.93	4.33
F9	1.53	1.67	4.37	6.70	4.70	4.27	4.77
F10	1.33	2.17	5.07	6.83	4.13	4.50	3.97
F11	4.37	1.23	4.37	6.47	2.80	5.63	3.13
F12	1.13	2.00	3.87	6.83	5.63	5.10	3.43
F13	2.43	3.53	4.43	6.67	2.50	5.83	2.60
F14	5.17	2.80	3.50	6.70	2.47	4.30	3.07
F15	2.43	3.83	3.37	6.77	4.03	4.50	3.07
F16	1.43	2.60	4.53	6.90	4.13	5.10	3.30
F17	1.67	1.60	4.70	6.87	4.27	5.30	3.60
F18	4.90	1.27	3.83	5.38	3.83	4.95	3.83
F19	4.67	1.23	3.92	5.53	3.78	4.95	3.92
F20	4.70	1.50	3.87	5.40	3.77	4.90	3.87
F21	1.67	1.63	5.03	6.57	3.43	5.47	4.20
F22	2.17	1.10	4.97	6.97	4.37	4.37	4.07
F23	1.37	1.77	5.17	6.67	3.90	5.07	4.07
F24	2.07	1.20	5.43	6.17	4.60	4.73	3.80
F25	1.17	2.70	4.07	3.80	5.70	5.93	4.63
Ave.	**2.13 **	**1.96 **	**4.55 **	**6.46 **	**4.06 **	**4.96 **	**3.88 **

	30-dimensional function						
F1	1.00	2.00	5.20	6.93	3.10	5.77	4.00
F2	1.00	2.87	4.77	6.97	2.70	5.77	3.93
F3	1.00	2.50	4.63	6.93	2.97	5.80	4.17
F4	1.03	3.33	3.97	6.87	2.57	5.80	4.43
F5	1.00	2.03	4.03	6.90	4.33	5.67	4.03
F6	1.00	2.00	5.47	6.90	3.50	5.43	3.70
F7	1.00	2.00	4.63	6.93	3.60	5.33	4.50
F8	1.00	3.93	4.37	6.77	4.27	3.60	4.07
F9	1.00	2.10	5.37	6.60	3.73	4.73	4.47
F10	1.03	1.97	5.23	6.87	4.90	3.90	4.10
F11	2.77	5.73	3.30	6.20	1.90	5.77	2.33
F12	1.00	4.80	2.80	6.33	6.20	4.37	2.50
F13	2.60	4.60	4.13	5.83	1.60	6.57	2.67
F14	3.93	5.67	2.90	6.20	2.67	4.80	1.83
F15	2.03	1.47	4.67	6.70	3.20	5.40	4.53
F16	1.53	1.70	4.37	6.80	4.40	5.33	3.87
F17	1.43	1.70	4.40	6.73	5.03	4.80	3.90
F18	5.00	6.30	2.02	3.95	2.02	6.70	2.02
F19	5.20	6.23	2.02	3.95	2.02	6.57	2.02
F20	5.17	6.33	2.02	3.95	2.02	6.50	2.02
F21	1.70	1.30	5.23	6.87	3.47	4.70	4.73
F22	1.07	1.93	4.30	6.97	4.27	4.87	4.60
F23	1.63	1.37	5.27	6.63	3.40	4.83	4.87
F24	1.37	1.63	5.10	6.30	4.67	3.77	5.17
F25	2.53	6.37	2.80	2.07	4.97	6.63	2.63
Ave.	**1.96 **	**3.27 **	**4.12 **	**6.17 **	**3.50 **	**5.34 **	**3.64**

**Table 7:** Algorithms' rank without DE by Friedman test for F1–F25 of 10D and 30D, respectively. The abbreviation meanings are in Table 2.

Function	Logistic	Tent	Gauss	Hénon-rand	Hénon-attrac.
	10-dimensional function				
F1	3.07	4.93	2.33	2.47	2.20
F2	3.17	4.87	1.73	2.73	2.50
F3	2.40	4.90	1.77	3.33	2.60
F4	3.30	4.60	2.03	2.50	2.57
F5	2.63	4.83	2.83	3.00	1.70
F6	3.20	4.77	2.50	3.03	1.50
F7	3.17	4.77	1.83	3.03	2.20
F8	2.33	4.73	2.57	2.97	2.40
F9	2.40	4.70	2.77	2.37	2.77
F10	3.13	4.83	2.20	2.57	2.27
F11	2.90	4.73	1.53	3.97	1.87
F12	1.93	4.83	3.63	3.10	1.50
F13	3.00	4.70	1.50	4.03	1.77
F14	2.67	5.00	1.73	3.27	2.33
F15	2.27	4.90	2.77	3.13	1.93
F16	2.67	4.90	2.47	3.23	1.73
F17	2.73	4.87	2.33	3.30	1.77
F18	2.47	4.02	2.47	3.58	2.47
F19	2.48	4.10	2.42	3.52	2.48
F20	2.50	4.03	2.43	3.53	2.50
F21	3.07	4.57	1.53	3.57	2.27
F22	3.00	4.97	2.43	2.43	2.17
F23	3.17	4.67	1.97	3.07	2.13
F24	3.43	4.17	2.70	2.77	1.93
F25	2.27	2.10	3.73	4.03	2.87
Ave.	**2.77 **	**4.58 **	**2.33 **	**3.14 **	**2.18 **

	30-dimensional function				
F1	3.20	4.93	1.10	3.77	2.00
F2	2.87	4.97	1.20	3.83	2.13
F3	2.67	4.93	1.33	3.80	2.27
F4	2.37	4.87	1.17	3.87	2.73
F5	2.07	4.90	2.33	3.67	2.03
F6	3.47	4.90	1.50	3.43	1.70
F7	2.63	4.93	1.60	3.33	2.50
F8	2.73	4.80	2.70	2.17	2.60
F9	3.37	4.60	1.73	2.77	2.53
F10	3.23	4.87	2.90	1.90	2.10
F11	2.70	4.67	1.50	4.33	1.80
F12	1.73	4.47	4.33	3.03	1.43
F13	3.03	4.23	1.07	4.73	1.93
F14	2.50	4.77	2.23	3.87	1.63
F15	2.73	4.70	1.50	3.43	2.63
F16	2.43	4.80	2.43	3.33	2.00
F17	2.43	4.77	3.03	2.80	1.97
F18	2.02	3.95	2.02	5.00	2.02
F19	2.02	3.95	2.02	5.00	2.02
F20	2.02	3.95	2.02	5.00	2.02
F21	3.23	4.87	1.47	2.70	2.73
F22	2.30	4.97	2.27	2.87	2.60
F23	3.27	4.63	1.40	2.83	2.87
F24	3.10	4.30	2.67	1.77	3.17
F25	2.27	1.60	4.00	5.00	2.13
Ave.	**2.66 **	**4.53 **	**2.06 **	**3.53 **	**2.22**

**Table 8:** Means of Gaussian mixture models with 3D, 5D, 7D, 10D, 13D, 15D, 17D, and 20 D. The abbreviation meanings are in Table 2. The (†) and (‡) marks mean that the proposed algorithms are significantly better than or as the same as IDE/best/1/bin and IDE/rand/1/bin, respectively, by Wilcoxon sign-ranked test (*P* < 0.05).

	DE-best	DE-rand	Logistic	Tent	Gauss	Hénon-rand	Hénon-attrac.
3D	−5.32	−5.05	− 4.81	− 4.96^†‡^	− 5.36^†‡^	− 4.86	− 5.08^‡^
5D	−2.47	−2.23	− 2.39^†‡^	− 2.52^†‡^	− 2.47^†‡^	− 2.47^†‡^	− 2.46^†‡^
7D	−1.50	−1.00	− 1.17^‡^	− 1.13^‡^	− 1.36^†‡^	− 1.25^†‡^	− 1.31^†‡^
10D	−0.34	−0.17	− 0.55^‡^	− 0.56^‡^	− 0.46^‡^	− 0.48^‡^	− 0.42^‡^
13D	−5.20	−5.17	− 4.73	− 4.98^‡^	− 5.32^†‡^	− 4.81	− 5.09^‡^
15D	−2.59	−2.13	− 2.49^†‡^	− 2.41^‡^	− 2.48^†‡^	− 2.43^†‡^	− 2.43^†‡^
17D	−1.32	−0.87	− 1.23^†^	− 1.24^†‡^	− 1.30^†‡^	− 1.28^†‡^	− 1.29^†‡^
20D	−0.35	−0.22	− 0.48^†‡^	− 0.46^†‡^	− 0.48^†‡^	− 0.38^†‡^	− 0.52^†‡^
